# Integrin β3 Reprogramming Stemness in HER2-Positive Breast Cancer Cell Lines

**DOI:** 10.3390/biology13060429

**Published:** 2024-06-11

**Authors:** Asiye Busra Boz Er

**Affiliations:** Department of Medical Biology, Faculty of Medicine, Recep Tayyip Erdogan University, 53020 Rize, Turkey; asiyebusra.bozer@erdogan.edu.tr

**Keywords:** HER2-positive breast cancer, integrin, Notch, stemness, trastuzumab resistance

## Abstract

**Simple Summary:**

HER2-positive breast cancer, treated with trastuzumab, often develops resistance within a year in about 50% of patients. This study identifies ITGβ3 as a key factor in promoting resistance by upregulating stem cell markers through the Notch signaling pathway. Combining trastuzumab with the integrin inhibitor cilengitide significantly reduces these markers, suggesting a potential therapeutic approach to overcoming resistance.

**Abstract:**

HER2-positive breast cancer, characterised by overexpressed HER2 levels, is associated with aggressive tumour behaviour and poor prognosis. Trastuzumab is a standard treatment; however, approximately 50% of patients develop resistance within one year. This study investigates the role of ITGβ3 in promoting stemness and resistance in HER2-positive breast cancer cell lines (HCC1954 and SKBR3). The findings demonstrate that chronic exposure to trastuzumab upregulates stem cell markers (*SOX2*, *OCT4*, *KLF4*, *NANOG*, *SALL4*, *ALDH*, *BMI1*, *Nestin*, *Musashi 1*, *TIM3*, *CXCR4*). Given the documented role of RGD-binding integrins in drug resistance and stemness, we specifically investigated their impact on resistant cells. Overexpression of ITGβ3 enhances the expression of these stem cell markers, while silencing ITGβ3 reduces their expression, suggesting a major role for ITGβ3 in maintaining stemness and resistance. Further analysis reveals that ITGβ3 activates the Notch signalling pathway, known for regulating stem cell maintenance. The combination of trastuzumab and cilengitide, an integrin inhibitor, significantly decreases the expression of stem cell markers in resistant cells, indicating a potential therapeutic strategy to overcome resistance. These results identify the importance of ITGβ3 in mediating stemness and trastuzumab resistance through Notch signalling in HER2-positive breast cancer, offering new approaches for enhancing treatment efficacy.

## 1. Introduction

Overexpressed HER2 (human epidermal growth factor receptor 2) levels are associated with 20–30% of breast tumours and are linked to poor prognosis [1]. Trastuzumab is the FDA-approved treatment for HER2-positive breast cancer, which decreases HER2 overactivation by multiple mechanisms, such as preventing HER2 dimerization, downregulating the HER2 receptor through endocytic destruction, and inhibiting the constitutive HER2 cleavage mediated by metalloproteases [2]. However, resistance remains a significant challenge, as approximately 50% of HER2-positive breast cancer patients develop resistance to trastuzumab within one year of treatment [3]. Resistance mechanisms include alterations in the HER2 receptor, such as mutations that prevent effective drug binding [4], activation of alternative signalling pathways, like the PI3K/AKT and MAPK pathways [5], and upregulation of other receptor tyrosine kinases (e.g., IGF-1R, MET) as well as enhanced HER2–HER3 dimerization that promotes cell survival and proliferation [6]. Additionally, the tumour microenvironment, including hypoxia, and the presence of cancer stem cells (CSCs), also promotes drug resistance [7,8].

The concept of “stemness” in cancer refers to the ability of cancer cells to exhibit characteristics similar to those of stem cells, such as self-renewal, differentiation potential, and resistance to conventional therapies [9]. This phenomenon is critical for tumour progression, metastasis, recurrence, and drug resistance. Previous research has demonstrated that CSCs contribute to the initiation and perpetuation of tumours and are often implicated in therapeutic resistance.

RGD-binding integrins which play a pivotal role in signalling recognize the Arg-Gly-Asp (RGD) motif present in various extracellular matrix proteins and modulate cancer stemness [10]. Integrins such as αvβ3 and αvβ5 are well-documented for their ability to influence CSC properties. For example, integrin αvβ3 has been shown to support the maintenance of stemness in glioblastoma and breast cancer cells, promoting tumourigenicity and resistance to therapy [11]. ITGβ3 has emerged as a significant player in cancer biology, particularly in the context of breast cancer. Recent studies have highlighted its role in reprogramming stemness in HER2-positive breast cancer cell lines, which is associated with aggressive tumour growth and poor prognosis [12]. 

Recent findings have shed light on the role of ITGβ3 in regulating stemness through various signalling pathways. In particular, ITGβ3 has been implicated in the activation of the Notch pathway, a critical regulator of cell fate determination, differentiation, and proliferation [13]. The Notch pathway is well-known for its role in maintaining stemness in normal and cancerous tissues; however, the ITGβ3–Notch association in many cancer types, including breast cancer, remains unknown.

In the context of HER2-positive breast cancer, the relation between ITGβ3 and the Notch pathway presents a novel axis of interest. The reprogramming of cancer cells towards a stem-like phenotype through ITGβ3 signalling and Notch pathway activation could explain the aggressive nature and treatment resistance observed in HER2-positive breast cancer. This study aims to understand the mechanism of integrin β3′s effect on stemness in HER2-positive breast cancer cells via the Notch signalling pathway. By analysing these molecular interactions, we aim to uncover potential therapeutic targets that could disrupt this axis, thereby inhibiting the stem-like properties of cancer cells and improving treatment outcomes for patients with HER2-positive breast cancer.

## 2. Materials and Methods

### 2.1. Cell Culture

HCC1954 (ATCC Cat#CRL2331) and SKBR3 (ATCC Cat#HTB30) are HER2-positive breast cancer cell lines purchased from ATCC. The cell lines were grown in DMEM media supplemented with 10% FBS, 1% sodium pyruvate, and 2 mM L-glutamine. The HCC1954 and SKBR3 cells were seeded into six-well plates (2.2 × 10^6^/well) at a confluency of 40–60% and transfected 24 h later with Lipofectamine 2000 according to the manufacturer’s instructions. 

### 2.2. Cell Viability Assay

The drugs were prepared with DMSO as 100 mM stock solution. Then, 200 µL cells (1 × 10^4^ cells/mL) were seeded in 96-well plates. The cells were treated in the range of 0.01 µM, 0.1 µM, 1 µM, 10 µM, 100 µM, and 1000 µM of the drugs. For blank wells, the solvent was used (DMSO) according to the maximum number of dilutions to exclude any effect of the solvent. All treatments were performed in triplicate and incubated for 96 h at 37 °C. The media were discarded, and the cells were incubated with MTT solution at a final concentration of 0.5 mg/mL. After 4 h, the MTT solution was removed, and formazan crystals were resuspended with DMSO. The absorbance was measured using a Multiscan Spectrum spectrometer at 550 nm (Thermo Fisher Scientific-Life Technologies Holdings Pte. Ltd., Singapore), and IC_50_ results were calculated with the Quest Graph™ IC50 Calculator [14].

### 2.3. Overexpression and Gene Silencing Experiments

pcDNA3.1-beta-3 was a gift from Timothy Springer (Addgene plasmid # 27289; http://n2t.net/addgene:27289 (accessed on 6 May 2024); RRID:Addgene_27289) [15]. Plasmids delivered with Lipofectamine 2000 (Invitrogen, Waltham, MA, USA) were incubated 48 h before analysis. A mock plasmid was used as the control with the same conditions.

The ITGβ3 mouse siRNA oligo (CAT#: SR427515) was purchased from Origene, delivered with Lipofectamine RNAiMAX (Invitrogen), and incubated 48 h before analysis. Scrambled RNA was used as the control with the same conditions.

### 2.4. Quantitative Real-Time PCR and Analysis

RNA was isolated using a Qiagen RNAeasy kit according to the manufacturer’s instructions. Biorad iScript Reverse Transcription Supermix for RT-qPCR was used for cDNA synthesis. PCR was carried out using the iTaq Universal SYBR Green One-Step Kit and Ct results were measured using the Applied Bioscience ABI 7500 Real-Time Instrument and 7500 Software v1. Amplification: (95 °C for 10 s, 60 °C for 1 min) ×40 cycle; melting: denaturation—95 °C for 15 s, annealing—60 °C for 1 min, elongation—95 °C for 15 s. Real-time PCR primers (*ITGB1*, *ITGβ3*, *ITGB5*, *ITGB6*, *ITGB8*, *ITGA2*, *ITGA2B*, *ITGA4*, *ITGA5*, *ITGAV*, *OCT4*, *Sox2*, *Klf4*, *Nanog*, *Sall4*, *Aldh*, *Bmi 1*, *Nestin*, *Musashi 1*, *Tim3 ve Cxcr’4*, *Gapdh*, *Hes*, *Hey*, *P21*, *Bcl1*, *Gata3*, *Ptcra*) were from Sigma KiqStart. Each sample and primer PCR were performed as triplicates as a technical repeat and the experiments were performed three times with different samples. To analyse RT-qPCR data, the cDNAs’ threshold cycle (Ct) values were normalised by the housekeeping gene GAPDH. Data were calculated according to the following formula: average of technical repeats and ΔCt = Ct (average of target gene) − Ct (average of housekeeping gene). The 2^–∆Ct^ value was calculated and 2^–∆∆Ct^:2^–∆Ct (sample)^/2^–∆Ct(control)^ was calculated to show fold changes. Statistical analysis was carried out using a two-tailed Student’s *t*-test. Differences were considered significant if * *p* ≤ 0.05. Error bars represent ± SD of three independent experiments, each conducted in triplicate. 

## 3. Results

### 3.1. Trastuzumab Resistance in HER2-Positive Breast Cancer Cell Lines Induces Stemness

**Figure 1:**
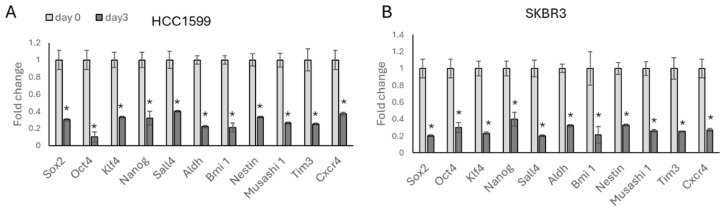
After a 3-day trastuzumab treatment, stemness-related gene expressions significantly decreased in HER2-positive breast cancer cell lines: (**A**) HCC1954 cells, and (**B**) SKBR3 cells. Student’s *t*-test, * *p* ≤ 0.05, n = 3 ± SD.

To analyse the effect of trastuzumab on HER2-positive breast cancer cells, MTT viability assays were performed with trastuzumab for the HCC1954 and SKBR3 cell lines, and the IC_50_ results of the cells were determined as 0.3 and 0.2, respectively (Table 1). The cells were treated with the IC50 level of trastuzumab for each cell line for 3 days, and a significant decrease in stemness-related transcription factors (*Sox2*, *Oct4*, *Klf4*, *Nanog*, *Sall4*) and stemness-responsive genes (*Aldh*, *Bmi1*, *Nestin*, *Musashi 1*, *Tim3*, *Cxcr4*) was observed for the HCC1954 (Figure 1A) and SKBR3 (Figure 1B) cell lines.

HER2-positive HCC1954 and SKBR3 trastuzumab-resistant cell lines were generated by exposing the cells to increased doses (0.1–10 μM) of trastuzumab for 3 months. To confirm the acquisition of resistance in the newly generated cells, MTT viability assays were performed, and the IC_50_ results of the cells were analysed. Chronic exposure to trastuzumab resulted in the development of resistance, with the cells exhibiting higher IC_50_ levels (Table 1).

**Figure 2:**
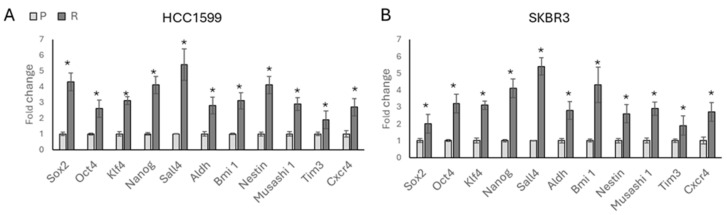
Acquired trastuzumab resistance is associated with increased expression of stemness-related genes in HER2-positive breast cancer cell lines. Significantly increased stemness-related gene expressions were observed after 3 months of chronic trastuzumab exposure in (**A**) HCC1954 cells and (**B**) SKBR3 cells. P: parental, R: resistant. Student’s *t*-test, * *p* ≤ 0.05, n = 3 ± SD.

Following confirmation of drug resistance using the MTT assays, we analysed the response of stemness marker expressions to the gained resistance. A significant increase was observed in the expression of stemness-related transcription factors, including *Sox2*, *Oct4*, *Klf4*, *Nanog*, and *Sall4*, as well as stemness-responsive genes such as *Aldh*, *Bmi1*, *Nestin*, *Musashi 1*, and *Tim3* in HCC1954 (Figure 2A) and SKBR3 (Figure 2B) trastuzumab-resistant cells. This suggests an association between acquired resistance and the upregulation of stemness markers in these cell lines.

### 3.2. Integrin B3 Has a Role in Stemness Reprogramming in HER2-Positive Breast Cancer Cell Lines

**Figure 3:**
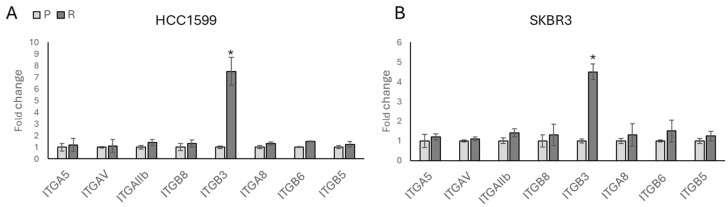
*ITGβ3* has a role in acquired trastuzumab resistance in HER2-positive breast cancer cell lines (**A**) HCC1954 and (**B**) SKBR3. P: parental, R: resistant. Student’s *t*-test, * *p* ≤ 0.05, n = 3 ± SD.

It is known that integrins play a crucial role in cell reprogramming and stemness and are also implicated in drug resistance in various cancers [12]. To investigate the relationship between RGD-binding integrins and trastuzumab drug resistance, we analysed gene expression levels. A significant increase was observed in *ITGβ3* levels, with a seven-fold increase noted in the HCC1954-R cell line (Figure 3A) and approximately a five-fold increase observed in the SKBR3-R (Figure 3B) cell line. However, no significant changes were detected in the levels of *ITGA5*, *ITGAV*, *ITGAIIb*, *ITGB8*, or *ITGB6* (Figure 3A,B).

**Figure 4:**
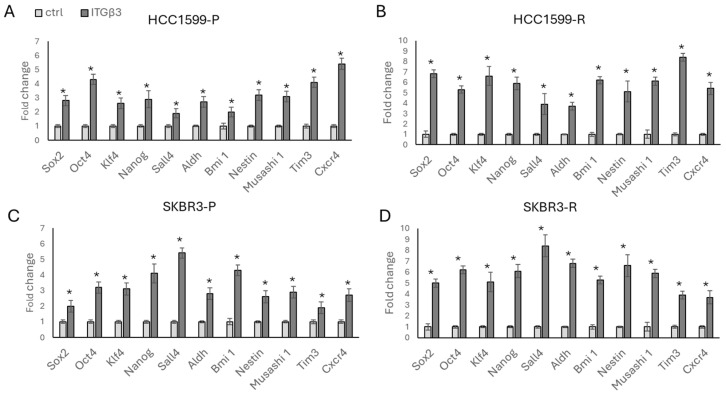
Overexpression of ITGβ3 increases stemness in parental and resistant cells. Significant increases were observed in stemness-related markers in the presence of ITGβ3 overexpression in (**A**) HCC1954-P, (**B**) HCC1954-R, (**C**) SKBR3-P, and (**D**) SKBR3-P cell lines. Light bars represent mock plasmid expression while dark bars represent ITGβ3 overexpression. P: parental, R: resistant. Student’s *t*-test, * *p* ≤ 0.05, n = 3 ± SD.

To investigate the impact of increased *ITGβ3* levels on drug resistance-associated stemness, we compared parental and resistant cells in the presence and absence of ITGβ3 overexpression (Figure 4) (see Supplementary Figure S1 for forced expression of ITGβ3). Subsequently, we analysed the expression levels of stemness-related markers. In both parental and resistant HCC1954 cells (Figure 4A,B), overexpression of ITGβ3 was associated with increased expression of stemness markers. This finding was confirmed in SKBR3 parental and resistant cells (Figure 4C,D), although the expression levels exhibited cell line-specific patterns. For instance, while *SALL4* expression has a two-fold increase in HCC1954-P cells (Figure 4A), a four-fold increase was observed in HCC1954-R cells (Figure 4A). Similarly, in SKBR3 cells, *SALL4* expression showed a five-fold increase in parental cells (Figure 4C), and an eight-fold increase in resistant cells (Figure 4D).

**Figure 5:**
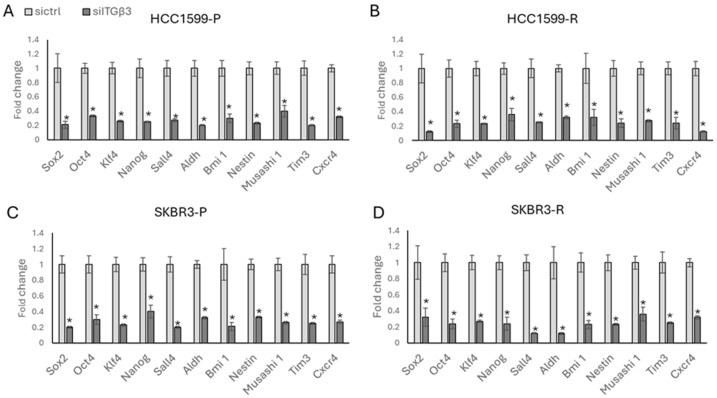
ITGβ3 silencing decreased stemness in HER2-positive parental and resistant cells. Silencing of ITGβ3 in (**A**) HCC1954-P, (**B**) HCC1954-R, (**C**) SKBR3-P, and (**D**) SKBR3-R decreased stemness-related markers. Student’s *t*-test, * *p* ≤ 0.05, n = 3 ± SD.

To understand the potential role of *ITGβ3* in reprogramming drug-resistant cells, we silenced ITGβ3 using siRNA (see Supplementary Figure S1 for the siITGβ3 western blot) in both the parental and resistant cells of HCC1954 and SKBR3, with scrambled RNA serving as a control. Silencing ITGβ3 in both the parental (Figure 5A) and resistant (Figure 5B) HCC1954 cells resulted in the decreased expression of stemness markers. This effect was confirmed in SKBR3 cells (Figure 5C,D), where the decreased expression of stemness markers was also observed upon ITGβ3 silencing in both parental and resistant cells.

### 3.3. Notch–ITGβ3 Axis Involved in HER2-Positive Breast Cancer Cell Lines

**Figure 6:**
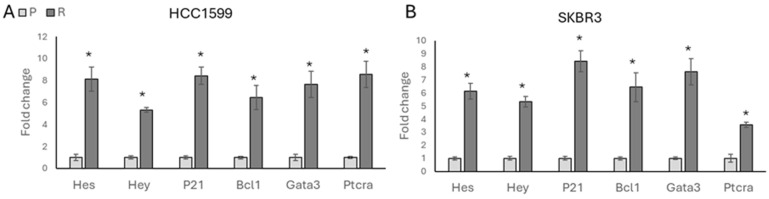
Drug resistance activates Notch signalling. A significant increase in Notch signalling-responsive genes was observed in (**A**) HCC1954 and (**B**) SKBR3 resistant genes. Student’s *t*-test, * *p* ≤ 0.05, n = 3 ± SD.

To understand the molecular mechanism associated with *ITGβ3* and stemness in drug-resistant cells, we investigated the association between ITGβ3 and the Notch signalling pathway. Initially, we analysed the expression of Notch-responsive genes and found a significant increase in resistant cells, indicating induced Notch pathway activity associated with acquired trastuzumab resistance in both HCC1954 (Figure 6A) and SKBR3 (Figure 6B) cells.

**Figure 7:**
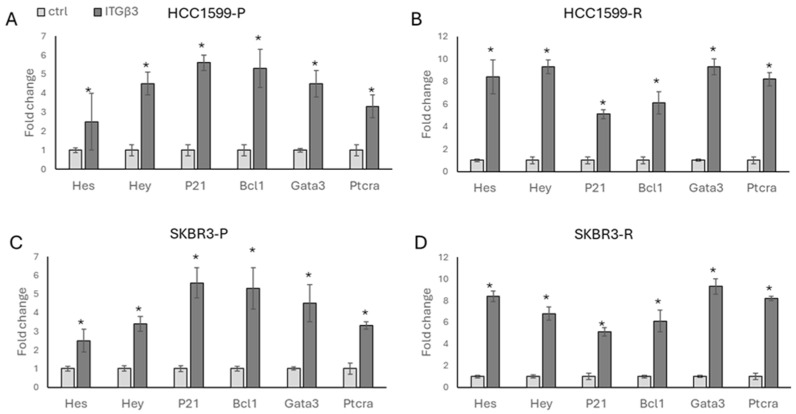
Overexpression of ITGβ3 induces Notch signalling in HER2-positive parental and resistant cells. A significant increase in Notch signalling-responsive genes was observed in (**A**,**B**) HCC1954 and (**C**,**D**) SKBR3 parental and resistant genes in the presence of ITGβ3. Student’s *t*-test, * *p* ≤ 0.05, n = 3 ± SD.

We overexpressed ITGβ3 in both parental and resistant HCC1954 and SKBR3 cells and analysed the expression of Notch-responsive genes. We observed increased expressions of *HES*, *HEY*, *P21*, *BCL1*, *GATA3*, and *PTCRA* in the presence of ITGβ3 overexpression in both parental and resistant cells of HCC1954 (Figure 7A,B) and SKBR3 (Figure 7C,D).

**Figure 8:**
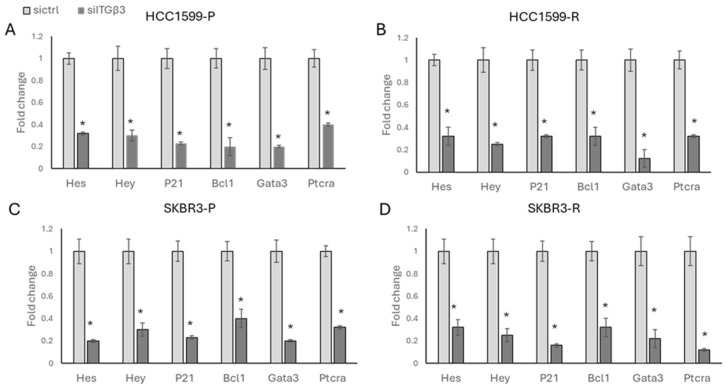
Silencing ITGβ3 decreases Notch signalling in HER2-positive parental and resistant cells. A significant decrease in Notch signalling-responsive genes was observed in both (**A**,**B**) HCC1954 and (**C**,**D**) SKBR3 parental and resistant cells upon ITGβ3 silencing. Student’s *t*-test, * *p* ≤ 0.05, n = 3 ± SD.

Additionally, we silenced ITGβ3 in both parental and resistant cells of HCC1954 (Figure 8A,B) and SKBR3 (Figure 8C,D) and observed a sharp decrease in *HES*, *HEY*, *P21*, *BCL1*, *GATA3*, and *PTCRA* levels. This suggests a regulatory role of ITGβ3 in modulating the Notch signalling pathway, thereby influencing stemness in drug-resistant cells.

### 3.4. Trastuzumab–Cilengitide Combination Decreases Reverse Stemness in HER2-Positive Breast Cancer Cell Lines

To observe the therapeutic potential of ITGβ3 in reprogramming the stemness of drug-resistant cells, we used cilengitide, an αvβ3 and α5β1 integrin inhibitor. Firstly, cilengitide sensitivity was analysed via an MTT assay, and IC50 values were calculated for HCC1954 and SKBR3 resistant cells (see Table 2).

**Figure 9:**
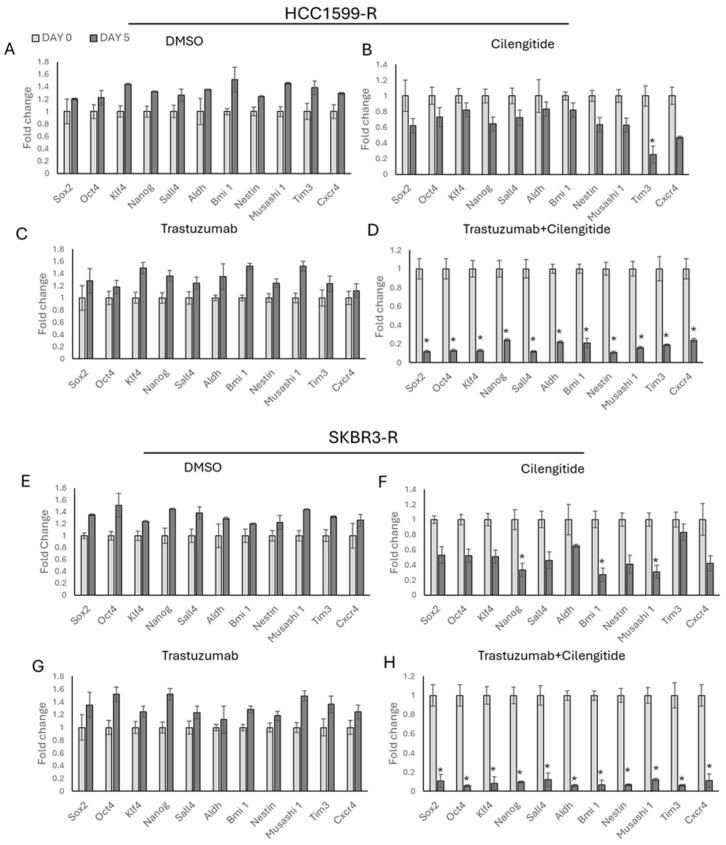
Trastuzumab–cilengitide combination decreased stemness markers, creating a synergistic effect in HCC1954 and SKBR3 trastuzumab-resistant cell lines. The resistant cells were treated for 5 days with monotherapy DMSO, trastuzumab, cilengitide, and the trastuzumab–cilengitide combination for (**A**–**D**) HCC1954 cells and (**E**–**H**) SKBR3 cells. Student’s *t*-test, * *p* ≤ 0.05, n = 3 ± SD.

Resistant cells treated with DMSO, trastuzumab, cilengitide as monotherapy, and trastuzumab with cilengitide for 5 days, as well as stemness marker expressions, were analysed. For trastuzumab monotherapy, the IC50 of the drug used for each resistant cell line is provided in Table 1, while for cilengitide monotherapy, the corresponding IC_50_ values are presented in Table 2. In the case of combination therapy, the IC_50_ of each drug used together is indicated. DMSO was used as a control and used as the maximum concentration of total drug in resistant cells. No significant response was observed in the presence of DMSO and trastuzumab (Figure 9A,C,E,G). However, for cilengitide monotherapy, each cell line demonstrated individual results. In HCC1954-R cells, cilengitide monotherapy decreased the *Tim3* stemness-related marker (Figure 9B), while in SKBR3-R cells, it affected *Nanog*, *Bmi1*, and *Musashi1*, but not *Tim3* (Figure 9F).

The trastuzumab + cilengitide combination created a synergistic effect, resulting in the decreased expression of *Sox2*, *Oct4*, *Klf4*, *Nanog*, *Sall4*, *Aldh*, *Bmi1*, *Nestin*, *Musashi1*, *Tim3*, and *Cxcr4* stemness-related markers in both HCC1954 and SKBR3 resistant cells (Figure 9D,H).

## 4. Discussion

The results of this study investigate mechanisms of trastuzumab resistance in HER2-positive breast cancer cell lines and propose potential therapeutic strategies for overcoming this resistance. The study highlights the role of stemness-related genes and *ITGβ3* in acquired trastuzumab resistance, proposing a combination therapy approach to reverse stemness which is promoted by resistance.

Chronic exposure to trastuzumab increases the IC_50_ values for both the HCC1954 and SKBR3 cell lines, confirming the development of resistance and mimicking a 2D cell culture model of HER2-positive breast cancer patients. This resistance correlates with elevated expression levels of stemness-related transcription factors (*SOX2*, *OCT4*, *KLF4*, *NANOG*, *SALL4*) and stemness-responsive genes (*ALDH*, *BMI1*, *Nestin*, *Musashi1*, *TIM3*, *CXCR4*). These markers, typically involved in maintaining self-renewing capabilities and the malignancy of cancer cells, suggest that trastuzumab-resistant cells acquire stem cell-like properties.

A sharp increase in *ITGβ3* expression is observed in resistant cells, with a seven-fold increase in HCC1954-R and a five-fold increase in SKBR3-R cells. Overexpression of ITGβ3 correlates with the increased expression of stemness markers in both the parental and resistant cell lines, while silencing ITGβ3 leads to a significant reduction in these markers. These results indicate that *ITGβ3* plays a crucial role in maintaining the stemness and drug resistance of HER2-positive breast cancer cells.

Further investigation reveals that the Notch signalling pathway is activated in trastuzumab-resistant cells, through increased expression of Notch-responsive genes (*HES*, *HEY*, *P21*, *BCL1*, *GATA3*, *PTCRA*). ITGβ3 overexpression enhances Notch signalling, while silencing ITGβ3 reduces Notch pathway activity. This suggests that ITGβ3 may drive stemness and resistance through the modulation of Notch signalling.

Given the role of ITGβ3 in trastuzumab resistance, cilengitide, an αvβ3/α5β1 integrin inhibitor, is investigated in combination with trastuzumab. While cilengitide monotherapy alone has varied effects on the stemness markers in resistant cells, its combination with trastuzumab leads to a synergistic reduction in stemness-related gene expressions. The combination therapy significantly reduces the expression of *SOX2*, *OCT4*, *KLF4*, *NANOG*, *SALL4*, *ALDH*, *BMI1*, *Nestin*, *Musashi1*, *TIM3*, and *CXCR4* in both HCC1954-R and SKBR3-R cells, suggesting that cilengitide can enhance trastuzumab efficacy and create a synergistic effect by targeting integrin-mediated stemness pathways.

## 5. Conclusions

These findings highlight the complexity of trastuzumab resistance in HER2-positive breast cancer and emphasize the involvement of stemness-related pathways and *ITGβ3*. Targeting ITGβ3, either through gene silencing or pharmacological inhibition using cilengitide, can disrupt the stemness characteristics and enhance the efficacy of trastuzumab. The combination therapy approach shows promise in overcoming trastuzumab resistance and improving outcomes for patients with HER2-positive breast cancer. Further clinical studies are necessary to validate these findings and optimize therapeutic strategies for resistant breast cancer.

## Figures and Tables

**Table 1:** Trastuzumab response of HCC1954 and SKBR3 cell lines. (n = 3 ± SD).

Cell Lines	P-IC_50_ (μM)	R-IC_50_(μM)
HCC1954	0.3 ± 0.05	2.4 ± 0.3
SKBR3	0.2 ± 0.04	2.6 ± 0.4

**Table 2:** Cilengitide response of HCC1954 and SKBR3 cell lines. (n = 3 ± SD).

Cell Lines	P-IC_50_ (μM)	R-IC_50_ (μM)
HCC1954	0.6 ± 0.12	0.7 ± 0.15
SKBR3	0.8 ± 0.14	0.6 ± 0.1

## Data Availability

The original contributions presented in the study are included in the article/Supplementary Materials, further inquiries can be directed to the corresponding authors.

## Supplementary Materials

The following supporting information can be downloaded at https://www.mdpi.com/article/10.3390/biology13060429/s1. Figure S1: Overexpression and silencing of ITGβ3 in HCC1954 and SKBR3 cell lines, (A) western blot and relative protein expression graph of overexpressed mock vector and ITGβ3 in SKBR3, (B) western blot and relative protein expression graph of sictrl and si ITGβ3 in SKBR3, (C) western blot and relative protein expression graph of overexpressed mock vector and ITGβ3 in HCC1954, (D) western blot and relative protein expression graph of sictrl and si ITGβ3 in HCC1954, quantitation was done by using ImageJ normalised with β-actin, results show average ± SD of three independent experiments.
