# Supplementary material for: Integrin β3 Reprogramming Stemness in HER2-Positive Breast Cancer Cell Lines
Source: Biology (Basel). 2024 Jun 11;13(6):429. doi: 10.3390/biology13060429 (PMC11201290; doi:10.3390/biology13060429)
Supplement: Supplementary file 1 [file biology-13-00429-s001.zip › biology-3039186-supplementary.pdf]

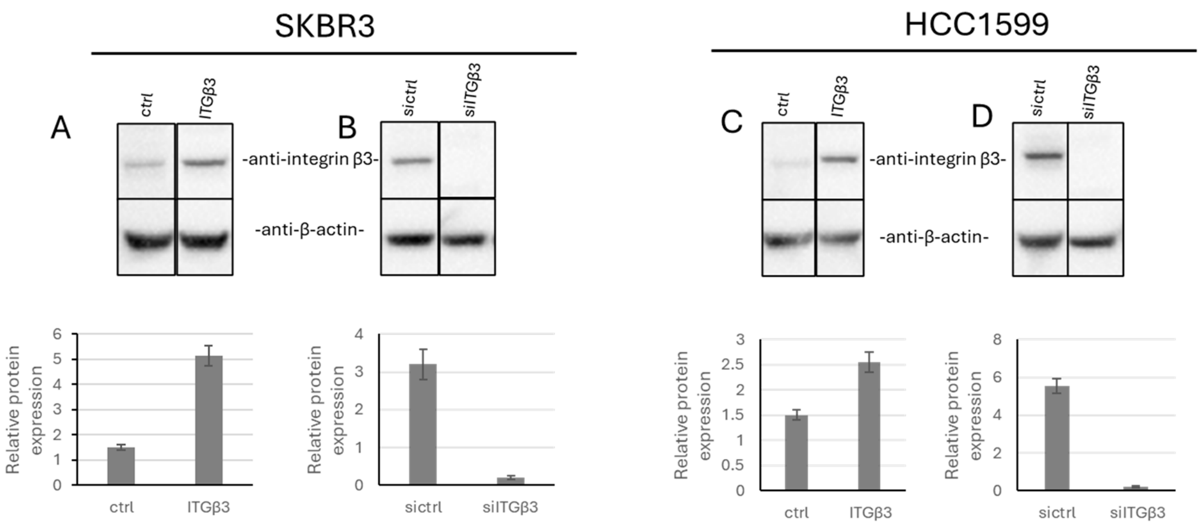

Supplementary Figure S1: Overexpression and silence of ITGβ3 in HCC1599, SKBR3 cell lines, A) western blot and relative protein expression graph of overexpressed mock vector and ITGβ3 in SKBR3, B) western blot and relative protein expression graph of sictrl and si ITGβ3 in SKBR3, C) western blot and relative protein expression graph of overexpressed mock vector and ITGβ3 in HCC1599, D) western blot and relative protein expression graph of sictrl and si ITGβ3 in HCC1599, Quantitation was done by using ImageJ normalised with β-actin, Results show average ± SD of three independent experiments
